# Yield instability of winter oilseed rape modulated by early winter temperature

**DOI:** 10.1038/s41598-019-43461-7

**Published:** 2019-05-06

**Authors:** James K. M. Brown, Rebecca Beeby, Steven Penfield

**Affiliations:** Department of Crop Genetics, John Innes Centre, Norwich Research Park, Norwich, NR4 7UH United Kingdom

**Keywords:** Plant breeding, Plant signalling

## Abstract

Yield stability is a major problem in oilseed rape with inter-annual variation accounting for between 30–50% of the crop value among the major global rapeseed producers. The United Kingdom has persistent problems with yield instability, but the underlying causes remain unclear. We tested whether temperature plays a role in UK winter oilseed rape (WOSR) yield variation through analysis of aggregated country-wide on-farm yield data and in annual Recommended List variety trial data run by the UK Agriculture and Horticulture Development Board (AHDB). Our analyses of the two independent datasets both show that mean temperature in early winter is strongly and uniquely linked to variation in WOSR yield, with a rise in mean temperature of 1 °C associated with an average reduction of 113 (+−21) kg ha^−1^ in yield. We propose that understanding the mechanism by which early winter chilling affects WOSR yield will enable the breeding of varieties with a more stable and resilient yield in Western Europe as climatic variation increases.

## Introduction

Yield stability is an important crop trait and determines the predictability of farm incomes on a global scale. Extreme environmental events such as heat, drought and flooding have important effects on global crop yields and are being exacerbated by environmental change^1^. However, even in the absence of extreme weather events inter-annual variation in yields can remain substantial. In oilseed rape normal variation in temperature and rainfall have important effects on yields^2–5^. In European winter oilseed rape (WOSR), yield variation caused by weather was much larger than that attributable to differences between cultivars in multisite trials^3^, and the genotype by environment (G × E) interaction was small. This indicates a lack of variation in yield stability traits in modern hybrids. Although advances in breeding have increased global rapeseed yields, this has not been accompanied by gains in yield stability^6^. For instance, in Europe increases in yield stability have only been found in environments that cause low overall yield^7^.

Several studies have linked developmental stages of rapeseed production with environmental variables such as light levels, photoperiod, temperature and rainfall, revealing those important for crop yields^8–12^. In central Europe temperature during seed filling is most strongly linked to yield^10^, whereas in Argentina, precipitation rates are limiting^11^. Studies at different sites therefore have the potential to uncover yield contingencies that may be masked at other sites through lack of weather variation at specific growth stages. Recent UK winter rapeseed harvests have shown high variability and thus it has become important to understand the mechanisms by which this yield variation occurs, and how this yield variation might be being affected by climate change.

To understand sources of yield instability in the UK rapeseed crop we sought to analyse the effects of temperature during 36 20-day windows of WOSR development. This analysis focusses on aggregated UK-wide yield data, and resulted in the identification of temperature windows with potentially important relationships with WOSR yield. The importance of highly correlated temperature windows was then tested in a separate dataset from 15 years of trials run by the UK Agriculture and Horticulture Development Board (AHDB). We show that across the two independent datasets, low temperatures at the end of November and beginning of December have a large association with UK rapeseed yields which is independent of genotype and trial location. Because low temperatures during this period are associated with higher yields, we conclude that December chill is a major determinant of UK rapeseed yields.

## Materials and Methods

### Aggregated UK winter rapeseed yield analysis

Aggregated UK-wide statistics for on-farm yields are published annually by the government Department for Environment, Food and Rural Affairs (DEFRA) for England, accessed on 08/02/2016 and 01/02/2018: https://www.gov.uk/government/statistical-data-sets/agriculture-in-the-united-kingdom. We chose to focus on data since 1990 because yield data prior to this time are marked by substantial rises and falls and because it coincides with widespread adoption of so-called ‘double low’ germplasm in the United Kingdom^13^. After 1990 steadily increasing on-farm yields over time can be approximated by a simple linear model. To approximate UK-wide mean temperature we used daily mean temperature data from the Met Office Hadley Centre Central England Temperature (HadCET) Series available at the NCAS British Atmospheric Data Centre, accessed on 08/01/2016 at the UK Met Office website^14^. The temperature dataset is generated with observations in Central England and thus is biased towards parts of the UK with greater land areas cultivated with WOSR. Sliding window analysis was conducted by averaging daily mean temperature values for 20-day periods beginning on the 1^st^, 10^th^ and 20^th^ of each calendar month for each WOSR growing season, starting in August and ending in July the following year. Winter North Atlantic Oscillation Index values were obtained from the Climate Research Unit at https://crudata.uea.ac.uk/cru/data/nao/ ^15^. Generalised Linear Modelling and Analysis of Variance were implemented in Genstat version 18 (VSN International).

### Agriculture and horticulture development board data analysis

The UK Agriculture and Horticulture Development Board runs annual Recommended List winter rapeseed trials. Harvest results for each variety at each trial location was available from 2002–2016 at the following URL: https://cereals.ahdb.org.uk/varieties/current-trials-and-harvest-results/archive.aspx. The data used here were Treated Seed Yields (t/ha) from trials where fungicides were applied to achieve near-complete disease control. The number of trials varies from year to year (Table S1). Data are reported as the yield (in tonnes ha^−1^) for each variety as a mean of two trial plots at each site. Yield data from all trials was collated resulting in a dataset with 6378 individual data points on yield, covering 15 years, 86 named trial sites and 252 varieties. Trial sites which were within 15 km of each other, in similar geographic situations and run by the same trialling company were grouped into locations for the purpose of data analysis (Table S1). We analysed two subsets of data. The main analysis was of yields from 21 trial locations (sites or groups of sites) used in at least five years and 29 varieties trialled over at least five years, giving a total of 1685 data points. An additional analysis included data from a total of 36 locations used in at least two years and all 251 varieties grown in those trials, with a total of 6314 values of yield. Data from 14 sites which were used in only one year and were not near another location were omitted from the analysis. Daily mean temperature data was not reported at all trial locations so we used the UK Meteorological Office MIDAS Land and Marine Surface Station Data UK daily temperature dataset (https://services.ceda.ac.uk)^16^. The closest Met Office monitoring stations were matched manually to individual trial sites. Statistical analysis was implemented in Genstat version 18, as described in the results.

## Results

**Figure 1:**
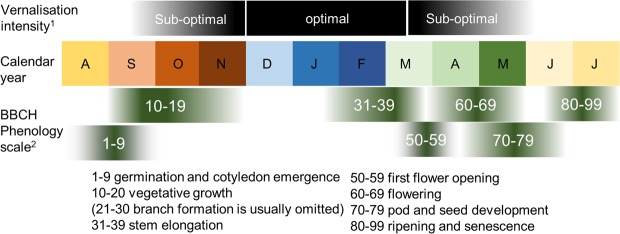
Phenology of winter oilseed rape in the UK. Vernalisation intensity and plant development of WOSR relative to the calendar year. 1 After Habekotté, 1997b. 2 as defined by Lancashire *et al*., 1991.

**Figure 2:**
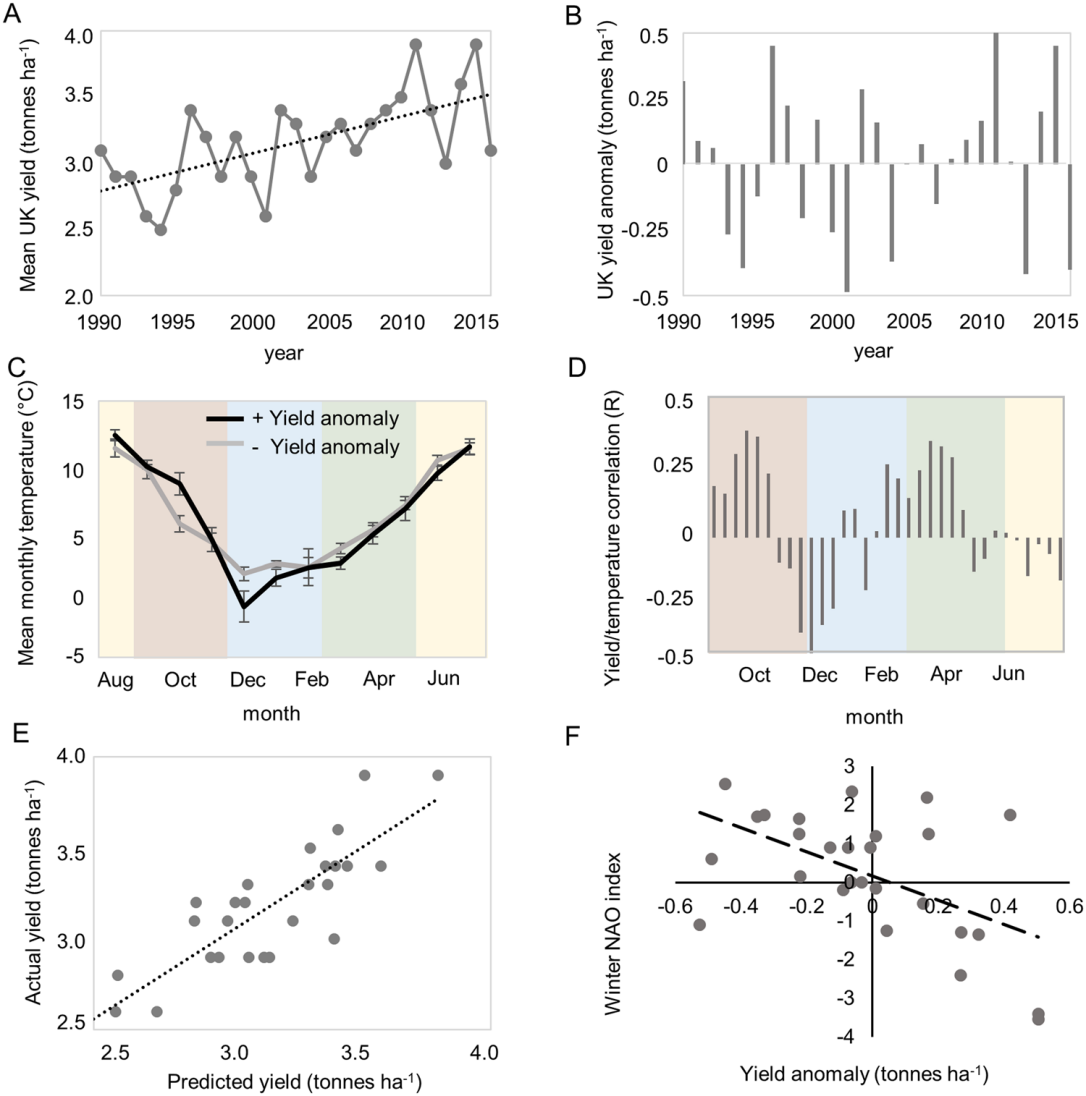
UK rapeseed yield is correlated with early December temperatures in the UK. (**A**) UK on-farm mean rapeseed yields from 1990, showing an increasing trend. (**B**) Rapeseed yield residuals from the best linear model in which yield is explained by year alone. (**C**) Comparison of the mean monthly temperatures in the 5 years with the strongest positive yield anomaly (black line), with the mean monthly temperatures in the 5 years with the strongest negative yield anomaly (grey line). Data are mean and standard error of UK on-farm yields for each calendar month. Colours indicate UK meteorological seasons from summer (yellow), autumn (brown), winter (blue) and spring (green). (**D**) Sliding window analysis, correlating mean temperature in 20 day windows starting on the 1st, 10th and 20th of each calendar month, for the period 1990–2015. Bars represent the correlation coefficient for each window. The window 1^st^–20^th^ December (P = 0.018) is not statistically significant after correction for multiple testing. (**E**) Generalised Linear Model output optimising the effect of mean temperature Dec 1^st^–Dec 20^th^ and year on UK mean aggregated rapeseed yield, and comparison to actual UK yields. (**F**) Relationship between UK WOSR yield and winter North Atlantic Oscillation (NAO) index (P = 0.004, n = 26) for the period 1990–2016. Yield anomaly was calculated as the deviation relative to a 10-year moving average, to account for variation in yield caused by genetic variation between varieties.

In the United Kingdom WOSR is drilled in late August, completes vegetative growth (BBCH 19)^17^ in November. After over-wintering the crop begins stem elongation (BBCH 31) in February and reaches BBCH51 (first flower open) in late March or April. Pod and seed development are completed by the end of June (Fig. 1). To examine the role of environmental temperature on rapeseed yield stability in the UK we began by analysing aggregated UK-wide on-farm yields from the period 1990 to 2016 (see methods). During this time mean WOSR yields have increased in the UK, in line with those of other countries^6^ (Fig. 2A). A simple linear model:

1 $$Y=constant+Year$$

whereby yield (*Y*) is related linearly to production year shows that year explains 25% of the variance in yield over this 26-year period (Table 1), showing the effect of advances in genetics and agronomy. UK rapeseed yield frequently deviates more than 0.4 tonnes ha^−1^ higher or lower than the predicted mean yield based on year alone (Fig. 2B), leading to an annual variability of up to 0.8 tonnes ha^−1^, or around 1/4 of the total harvest. Furthermore, the frequency of large deviations from the mean yield has been high since 2010. A key novel feature of North European weather patterns in the last decade has been an increase in more extreme temperature events caused by the response of the jet stream to climate change, in particular to the loss of Arctic sea ice^18,19^. Hence, we focussed on the role of temperature and tested the hypothesis that temperature during key stages of rapeseed crop development might explain yield variation.

**Table 1:** GLM analysis of the relationship between mean UK on-farm WOSR yield and mean HadCET temperature.

Variable	Bbch scale	Df	Yield gain/loss per 1 °c temperature rise (kg ha^−1^), or per year+− se	Significance
Year	n/a	1	21 +/− 3	P < 0.001
Dec(1–20)T_m_	20–29	1	111 +/− 19	P < 0.001
Oct(1–20)T_m_	14–19	1	79 +/− 25	P = 0.001
Apr(1–20)T_m_	60–69	1	70 +/− 35	P = 0.059
**Removing Apr**				
Year	n/a	1	17.6 +/− 5.0	P < 0.001
Dec(1–20)T_m_	20–29	1	−106 +/− 20	P < 0.001
Oct(1–20)T_m_	60–69	1	93.5 +/− 26	P = 0.002

The contribution of annual windows with high sensitivity (Fig. 1D) beginning on 1^st^ December (Dec), October (Oct) and April (Apr) to yield variation is shown. T_m_: mean temperature. The data show that growing season (harvest year) and DecT_m_ together explain 74% of the variance of the dataset, whereas OctT_m_ has a smaller but still significant effect.

For a preliminary test we compared the mean monthly temperature for the 5 years with the strongest positive yield deviations from the mean yield predicted by year alone (Eq. (1)), with the 5 years with the strongest negative deviation (Fig. 2C). This revealed that high yielding years were associated with warmer temperatures in October and colder temperatures in December, relative to the lowest yielding years. October corresponds to BBCH growth stages 11–19 and December to the period of growth cessation separating BBCH19 from BBCH31 (Fig. 1; note that BBCH stages 20–30 are usually omitted from the standard rapeseed phenology description)^17,20^. To further analyse this effect, we performed a sliding window analysis, analysing the correlation between mean WOSR yield and mean temperature in 36 annual 20-day windows for the UK WOSR growing season, beginning on the 10^th^ August until 20^th^ July the following year, with windows starting on the 1^st^, 10^th^ and 20^th^ of each month. Correlation coefficients relating to mean window temperature 1990–2016 and for each window are shown (Fig. 2D). This revealed weak associations with windows starting in October, late November/ early December and late March/ April. The only individual association that was significant was the one starting on December 1^st^ (P = 0.018), although this was not considered significant when a range of multiple testing procedures were applied. As associations with single variables have limited value for analysis of complex datasets we modelled mean yield (Y) as the sum of the effects of variables *Year*, mean monthly October temperature (*OctT*_*m*_), mean temperature December 1^st^–20^th^ (*DecT*_*m*_) and mean monthly April temperature (*AprT*_*m*_):

2 $$Y=a+b.Year+c.DecTm+d.OctTm+e.AprTm$$

where a–e are parameters estimated by the model. Both December and October temperature had a significant relationship with yield, whatever the order in which the different monthly temperatures were added to the model (Table 1). December temperature in particular had a strong negative correlation with yield, and December temperature and year explained 60% of yield variation in the dataset (Fig. 2E). There was a negative correlation between October and December temperatures (P = 0.01) implying that these effects are partially aliased.

Winter temperature in the UK is strongly affected by the North Atlantic Oscillation (NAO; 19), and recent variation in the mean temperature between December 1^st^–20^th^ correlates with the winter NAO index for the corresponding year (Fig. 2F)^15^. Therefore, December temperature is tightly associated with UK oilseed rape yield variation, and is under the influence of the NAO, and aliasing between the positive effects of October warmth and December cold is likely because both are influenced in opposite directions by the NAO^14^.

**Figure 3:**
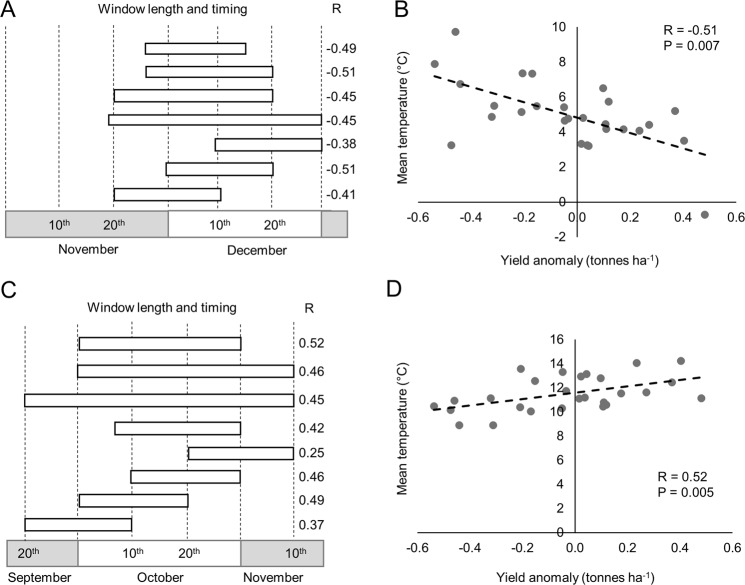
Defining the optimum window duration and start date for maximising the association strength between yield and mean temperature windows. (**A**) Optimisation of the duration and start date if the December window Data shows R values for the correlation between the HadCET mean temperature and UK mean yield anomaly for each time period indicated for 26 growing seasons starting in 1989/1990. (**B**) Scatter plot to show the relationship between mean temperature in the December window and UK WOSR yield anomaly for the period 1990–2016. (**C**) Optimisation of the duration and start date if the October window. Data shows correlation coefficient for the relationship between the HadCET mean temperature and mean UK yield anomaly for each time period indicated for 26 growing seasons starting in 1989/1990. (**D**) Scatter plot to show the relationship between mean temperature in the October window and UK WOSR yield anomaly for the period 1990–2016.

Next we manually optimised the window length and start dates of the October and December temperature windows by comparing mean temperature in different windows close to Dec. 1–20^th^ and the correlation with yield (1990–2016). For the December window this revealed that the coefficient of determination (R) was maximised during a window that began on 27^th^ November, and ended close to 21^st^ December (Fig. 3). This corresponds to the period after which vegetative growth has ceased (BBCH19). In the case of October, the period from 1^st^ to 31^st^ October had the highest R (0.25; Fig. 2). This corresponds to the period of vegetative growth that begins after seedling establishment (BBCH 14–19). We therefore used two time-periods, 1^st^–31^st^ October, and 27^th^ Nov to 21^st^ Dec in a further study to test the hypothesis that environmental temperature is a determinant of UK WOSR yields.

**Figure 4:**
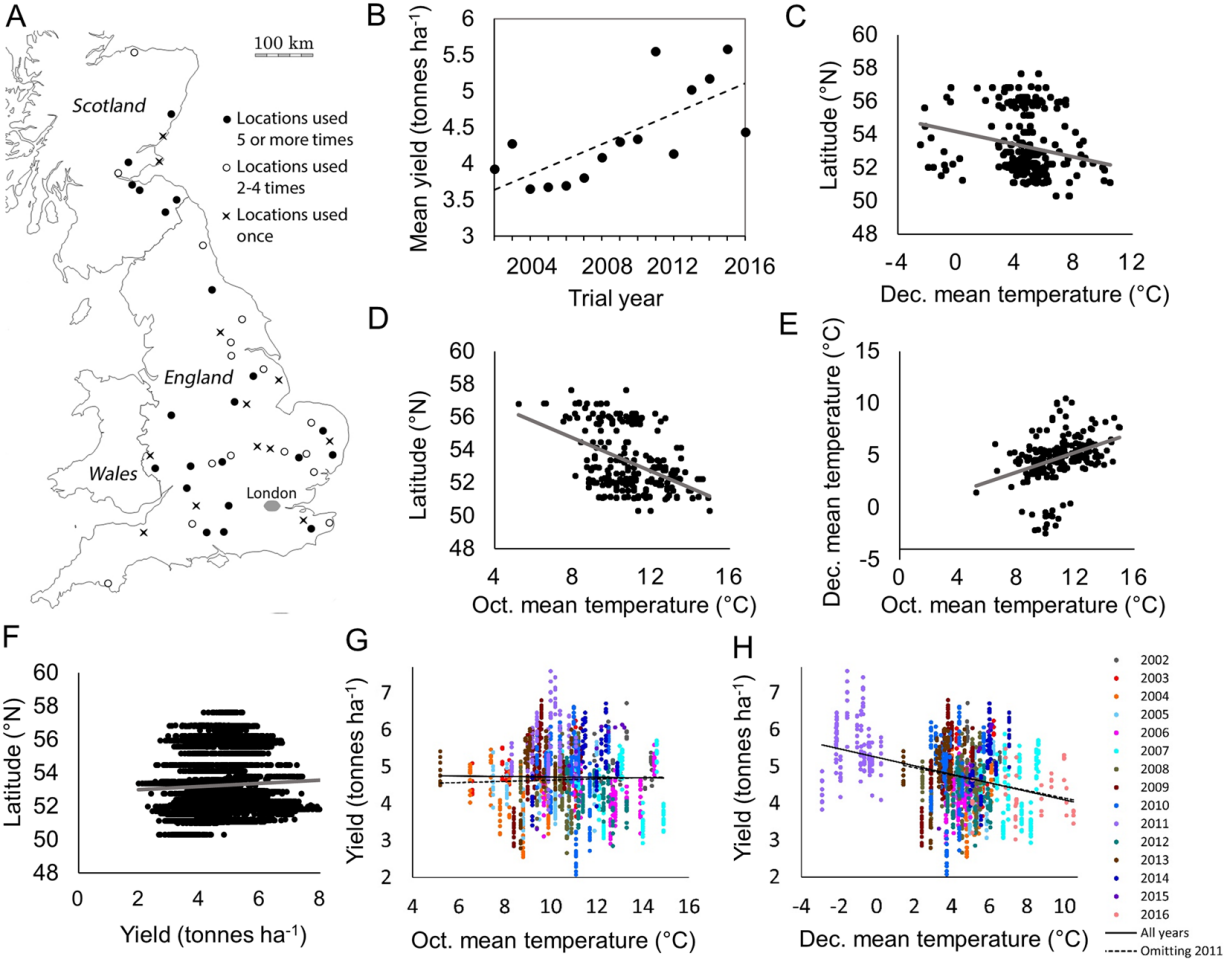
WOSR yield relationships in the AHDB National List trials dataset. (**A**) Outline map of the United Kingdom showing locations of AHDB Recommended List Trial sites 2002–2016. Map was created in Microsoft Powerpoint for Office 365 MSO (https://products.office.com/en-gb/powerpoint). (**B**) Plot to show the mean annual trial yield of the NL trials, which increases with time. (**C**–**H**) Scatter plots of individual variety yield measurements with linear line of best fit illustrating relationships between yield, temperature in December (Nov 27^th^–Dec 21^st^; Dec) and October (Oct), and trial latitude. Datapoints represent trial mean yield (**C**–**E**) or variety mean yield (**F**–**H**). For (**H**), datapoints with the same colour represent yield measurements in the same year (2002–2016).

We conducted this test using an independent dataset from the UK Agriculture and Horticulture Development Board, which runs Recommended List (RL) trials annually, collecting data for candidate WOSR varieties at up to 16 trial sites across the UK (Fig. 4A). Data were available for the years 2002–2016 (Table S1). In the RL trials, mean yield also increased on average between 2002 and 2016, as in the aggregated national data (Fig. 4B), and the national on-farm dataset and RL trial dataset shared features such as increased variation in yield measurements in later years (Figs 1A,B, 4B). A key difference between the two datasets is that because the RL trial data is listed by variety, we can separate yield variation achieved through breeding from that which results from other factors. Thus we assembled a dataset with 6378 individual yield measurements, using 21 trial locations used at least twice across 15 years and omitting 415 data points from isolated trial sites used only in one year. Each trial was associated with a mean October and mean ‘December’ temperature, with December referring to the period between November 25^th^ and December 21^st^ for that WOSR growing season (see methods). Initial analysis showed that October and December temperature were cooler at higher latitudes, and thus we could observe a positive relationship between October and December temperature (Fig. 4C–E). However, there was no clear relationship between latitude and mean trial yield (Fig. 4F), despite the fact that lower temperatures appeared to be associate with higher yields (Fig. 4G,H).

We selected a subset of the AHDB dataset containing only varieties and trial locations used in at least 5 of the 15 years. This resulted in the selection of 1685 data, including a total of 29 varieties grown at 21 Trial locations; up to 19 varieties were grown at each location in each year. In the data analysis, we sought to distinguish the effect of temperature on variation in WOSR yield from other factors, including the introduction of new varieties, which normally have higher yield than existing varieties, and variation between trial locations. The mean yield at each location in each year was estimated and its relationship to temperatures at critical periods determined.

We fitted a linear mixed model to Yield (*Y*) with four fixed effects: Trial location [*TL*], December mean temperature [*DecTm*], October mean temperature [*OctTm*] and the linear effect of year [*YearL*], and three random effects: varieties [*Var*], the sensitivity of each variety to December temperature [*Var*.*DecTm*], and specific effects of each trial location each year [*TL*.*Year*]. Location was treated as a fixed effect because the trial sites used each year are chosen to represent different, specific WOSR growing conditions, whereas variety was treated as a random effect because actual varieties can be considered to be a sample of all the possible elite genotypes of WOSR. The effects of *YearL* and *OctTm* were not statistically significant at the *P* < 0.05 level (*P* = 0.4 and 0.1 respectively) and were successively dropped from the model. The following model was therefore used to calculate the predicted mean yield at each location used in each year:

3 $${\rm{Fixed}}\,\mathrm{Model}:Y=constant+[TL]+[DecTm]$$

4 $${\rm{Random}}\,\mathrm{model}:Y=[Var]+[Var.DecTm]+[TL.Year]$$

Examination of a range of models indicated that the linear effect of year on increasing yield was largely explained by the introduction of new, successively higher-yielding varieties. This accounted for the lack of a significant effect of *YearL* in the model when *Var* was included as a random effect.

There was a strong effect of *DecTm* on the mean yield at trial locations (Table 2), with a 1 °C rise in temperature during this period corresponding to a mean yield decrease of 116 kg ha^−1^ across all varieties and trial sites (95% confidence interval: 70–162 kg ha^−1^). All trial locations with *DecTm* < 1 °C were in 2011 and all locations in 2011 had *DecTm* < 1 °C. We therefore repeated the analysis omitting all data from 2011, in case the effect observed was unduly influenced by that year’s data. The strong correlation between *DecTm* and mean yield remained, with a rate of decline of yield with increasing temperature of 104 ± 38 kg ha^−1^ K^−1^ (*P* = 0.007 for the *DecTm* effect).

**Table 2:** Linear Mixed Model analysis of the relationship between mean trial temperature (25^th^ Nov–21^st^ Dec; DecT_m_2), trial site, variety and WOSR yield.

Predictor variable	d.f.	F statistic	Estimate (kg ha^−1^ °C^−1^) ± SE	Significance
**Locations & varieties used in at least 5 years** (**1685 yield data**)				
*Location*	20, 140	5.09		P < 0.001
*YearL*	1, 153	0.72	−13 ± 15	P = 0.4
*OctTm* (*BBCH14–19*)	1, 141	2.55	57 ± 36	P = 0.1
*DecTm* (*BBCH* (*20–29*)	1, 156	26.46	−127 ± 25	P < 0.001
***YearL*** **and** ***OctTm*** **removed from model**				
*Location*	20, 142	5.04		P < 0.001
*DecTm*	1, 158	24.12	−116 ± 24	P < 0.001
**Locations used in at least 2 years** (**6378 yield data**)				
*Location*	36, 170	4.40		P < 0.001
*YearL*	1, 177	0.00	1 ± 14	P = 1.0
*OctTm* (*BBCH14–19*)	1, 171	1.48	39 ± 32	P = 0.2
*DecTm* (*BBCH* (*20–29*)	1, 179	28.78	−122 ± 23	P < 0.001
***YearL*** **and** ***OctTm*** **removed from model**				
*Location*	36, 172	4.41		P < 0.001
*DecTm* (*BBCH* (*20–29*)	1, 182	27.80	−113 ± 21	P < 0.001

The contributions of fixed effects are shown. *Location*: trial sites or groups of sites (Table S1). *YearL*: the linear effect of year fitted as a numerical variable. Random effects were *Variety* + *Variety*.*DecTm* + *Location*.*Year* in all models; in the latter case, *Year* is fitted as a factor to explain year-specific variation at trial locations. n/a: not applicable.

The lack of a significant effect of *OctTm* on yield in the RL trials (Table 2) led us to revisit the association between UK farm yields and October temperature for the period 2002–2016. Interestingly, no significant relationship was observed in on-farm harvest data for this time period, whereas for the period 1990–2003 a much stronger positive association was clear (Fig. S1). Therefore, rapeseed yield relationships with October temperature are not stable over time in UK on-farm data, and currently are not associated with yield variation. Furthermore, we cannot rule out that a link between October temperature and yield appears only when October mean temperature is negatively-correlated with December mean temperature (Fig. S1), which would indicate that the October temperature has a correlative rather than causal relationship with yield.

Trial location had an important relationship with yield (P < 0.001), with the highest-yielding location, Wardington, yielding on average 2042 kg ha^−1^ higher than the lowest-yielding site, Owmby-by-Spital. There was little evidence for variation between locations in the size of the effect of *DecTm* on yield; examination of a range of models indicated that while an interaction between *TL* and *DecTm* cannot be excluded, it is dwarfed by other factors which caused yields at individual trial sites to vary between years encompassed in the *TL*.*Year* term. As expected, Variety had a very strong effect on yield; the Akaike Information Coefficient (AIC) was lower by 178 when *Var* was included as a random effect than when it was not (a reduction in AIC of 2 is generally considered to indicate that a random effect contributes significantly to a mixed model).

Varieties varied in the extent to which their yields declined with increasing December temperature (AIC reduced by 41 when *Var*.*DecTm* included as random effect). While all varieties had lower predicted mean yields at higher temperatures, the gradient of the decline ranged from 155 kg ha^−1^ K^−1^ for the most responsive variety, DK Cabernet, to 59 kg ha^−1^ K^−1^ for the least responsive, Castille (Fig. S2). The mean effect of varieties’ response to temperature, calculated from the *Var*.*DecTm* term in the mixed model, was not correlated significantly with the predicted mean yield of the varieties (*P* = 0.5).

**Figure 5:**
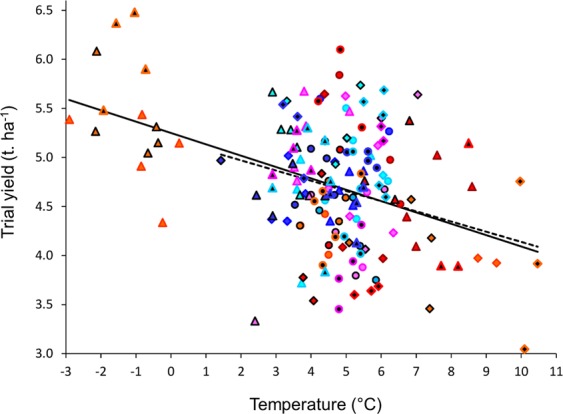
The relationship between mean temperature (Nov 27^th^–Dec 21^st^) and RL trial yield. Data are from trial locations and varieties used for at least five years. Each point is the mean yield at a location in one year, adjusted for changes in the use of varieties and for variation in yield between locations that was not explained by December temperature. AHDB regions and years are indicated by different symbols. The model relating yield to December temperature was fitted using Eqs 3 and 4. It is robust when data from harvest year 2011, when winter was unusually cold, are omitted (dashed line). The trend is apparent throughout Great Britain (AHDB regions used for variety recommendation: East, six locations in the East Midlands, East Anglia and Kent; North, seven locations in Scotland and northern England; West, eight locations in western England, Oxfordshire and Hampshire).

We also tested a similar model with data from all locations used in two or more years and all varieties trialled at five or more locations, including a total of 6378 yield data. As before, the linear effect of year and *OctTm* were not statistically significant (*P* = 1.0 and 0.2 respectively), and were therefore successively omitted from the model. As before, *DecT*_*m*_ was again highly negatively associated with yields for trials and varieties, with a 1 °C fall in temperate resulting on average in a 113 kg ha^−1^ gain in yield (95% CI: 71–155 kg ha^−1^ K^−1^; Table 2; Fig. 5). As before, the negative correlation between mean yield and *DecTm* continued to be strong when the 2011 data were omitted, with a yield decline of 108 ± 32 kg ha^−1^ K^−1^ (*P* < 0.001 for the *DecTm* fixed effect). This negative relationship between *DecT*_*m*_ and yield was comparable to that obtained in the independent UK aggregated farm data, with low temperatures associated with high yield for that cropping season (Fig. 4E). Therefore WOSR yield in the UK is correlated with chilling during the period of growth cessation which separates growth stages BBCH19 from BBCH31.

To test whether temperature in December is significantly aliased with other meteorological variables likely to affect yield in the UK, we analysed data for temperature, days of frost, rainfall and sunshine hours at 12 weather stations from the major UK winter rapeseed growing regions during the study period (2002 and 2016). This showed that temperature correlated strongly with days of frost, but not with sunshine hours or total rainfall (Fig. S4). The latter is unsurprising as soil moisture levels are high in the UK during winter^21^. Taken together, our analysis shows that chilling during formation of side shoots has a strong positive relationship with UK WOSR yields, a relationship visible both in aggregated on-farm data, and in expert-led AHDB Recommended List trials.

## Discussion

Yield stability is a major concern for food policy because fluctuations in crop production lead to uncertainty in farm incomes and thus to variability in consumer prices. It is a particular problem for the UK WOSR harvest leading to a decline in WOSR crop area since 2012. With on-farm yields showing inter-annual variation of up to 0.8 tonnes ha^−1^ (Fig. 2A), understanding the factors that cause yield variation could lead to improvements in predicting yield variation and allow us to understand how climate change is affecting WOSR yields.

We tested the effects of temperature variation on UK rapeseed yields, and found strong correlations with temperature during October and even more so, the first 20 days in December for time-series including 25 years starting in 1990 (Fig. 2; Table 1). However, in a second larger dataset from 2002 to 2016 we could confirm the strong effect of December temperature, but not the effect of October (Fig. 4; Table 2). The important period from late November through December coincides with the period of growth cessation which separates growth stages BBCH19 from BBCH31, after the completion of vegetative development but before elongation of the inflorescence begins. During this period the early developmental events that determine canopy characters occur, and canopy area is known to be highly correlated with WOSR yield^22^. Analysing the same time window in the national dataset also does not yield a strong correlation with October mean temperature, suggesting that temperature during this period has become less associated with yields in recent years (Fig. S1). This analysis resembles that observed for wheat grain characters in the long term Broadbalk experiment, where a positive relationship between NAO and Hagberg falling number was stable over short timescales, but varied in decadal timescales^23^. The effect of December temperature variation was to reduce yields by 78 kg ha^−1^ in the UK farm yield dataset and 116 kg ha^−1^ in the Recommended List field trials for each 1 °C increase in mean temperature. The difference in the size of the effect probably reflects the nature of crop management in the two situations, because RL Treated Yield trials aim to reveal varieties’ yield potential under optimal crop management. Therefore, improvements in agronomy on UK farms using current knowledge may increase yields, but is unlikely to improve yield stability. Scaling for UK cropping area and price, on-farm yield variation attributable to December temperature has led to large fluctuations in national income from the rapeseed harvest, with the gap between the lowest and highest income years exceeding £150 million. Previous work has identified a link between photosynthetically active radiation during pod filling and yield^10^. Here we show that the effects of chilling are also associated with high yields.

Temperature in December period correlates strongly with the NAO index^15,24^, suggesting that the NAO is a major driver of UK winter rapeseed yields. Indeed, we found that winter NAO index is negatively-correlated with the yield anomaly, i.e. the deviation of WOSR yield from its 10-year average (Fig. 2F). This is consistent with known effects of the NAO on phenology in natural ecosystems in Northern Europe, where the NAO explains between 9 and 28% of variation in flowering dates and 63% of variance in boreal phenology^25^, invertebrate population behaviour^26,27^ and fish spawning time^28^. This has implications for future yields because climate change is interacting with the NAO to increase variation in mid-latitude temperatures during winter^29^.

There is some variation between WOSR varieties in the extent to which their yields fall as mean temperature during the critical period of December rises. The variety with the strongest yield response to temperature, DK Cabernet, was predicted to have a difference in yield of 2,083 kg ha^−1^ between the years with the highest and lowest mean December temperatures (10.47 °C and −2.9 °C respectively; mean yield predicted across sites) while the least responsive variety, Castille, was predicted to have a yield difference of only 789 kg ha^−1^. From these results, we predict that substantial, unforeseen yield losses may be caused by warmer Decembers in highly temperature-responsive varieties. The lack of a significant correlation between varieties’ responses to temperature and their predicted mean yield across years and sites indicates, however, that the two traits are not necessarily under common genetic control (Fig. S3), so it should be possible for breeders to select varieties with high, stable yields by trialling their genotypes at a range of sites with diverse environmental conditions, particularly temperature in early winter.

The reason that temperature during a short early winter time period has a major association with yield in winter rapeseed remains unclear. Previously it has been suggested that cold periods could supress the effects of pests or pathogens^30^, but no specific effect has been proposed, and such a theory does not explain why cold only in this period has the yield-promoting effect, rather than cold later in winter. In our view these effects are unlikely to be mediated by agronomy, because RL trials are always substantially superior in agronomy and yield outcomes than commercial farms, yet are subject to similar inter-annual yield instability. In wheat the winter NAO has been shown to be associated with grain quality, including specific weight and Hagberg Falling Number^22,31^. Thus winter weather is known to have strong associations with yield and grain quality components in other species.

In Europe it has been predicted that climate change will affect rapeseed growth by limiting water availability, particularly in France, Germany and the United Kingdom by 2030^32^. Our study shows that extremes of warmth in December are also impacting farm incomes, and further work will be required to understand how future variation in December temperatures in Europe will affect WOSR crop values.

## Conclusions

Our study shows that temperature variation in late autumn and early winter is associated with yield instability in WOSR harvests in the United Kingdom, with lower temperatures between 27^th^ November and 21^st^ December correlated with higher crop yields at harvest. Further work is required to determine if this is due to the effects of weather on crop development, or on other factors that may influence WOSR yield. Warmer temperatures can also negatively affect semi-winter rapeseed yields in some regions of China^33^, so the implications of this study may impact other oilseed rape growing regions with unreliable winter chill. Further work will be needed to uncover whether yield variation is likely to known chilling responses in winter annuals, such as vernalisation requirement, play a role in this yield variation.

## Supplementary information

Supplementary Figures and Table

## References

[1] Lesk C, Rowhani P, Ramakutty N (2016). Influence of extreme weather disasters on global crop production. Nature.

[2] Sidlauakas G, Bernotas S (2003). Some factors affecting seed yield of spring oilseed rape (Brassica napus L.). Agron. Res..

[3] Nowosad K, Liersch A, Poplawska W, Bocianowski J (2016). Genotype by environment interaction for seed yield in rapeseed (*Brassica napus* L.) using additive main effects and multiplicative interaction model. Euphytica.

[4] He D, Wang E, Wang J, Lilley J (2017). Genotype × environment × management interactions of canola across China: A simulation study. Agric. Forest Meteorol..

[5] Robertson MJ, Lilley JM (2015). Simulation of growth, development and yield of canola (*Brassica napus*) with APSIM. Crop Pasture Sci..

[6] Rondanini DP, Gomez NV, Belen Agosti M, Mirallas DJ (2012). Global trends of rapeseed grain yield stability and rapeseed-to-wheat yield ratio in the last four decades. Eur. J. Agron..

[7] Peltonen-Sainio P, Jauhiainen L, Hannukkala A (2007). Declining rapeseed yields in Finland: how why and what next?. J. Agric. Sci..

[8] Habekotté B (1997). Identification of strong and weak yield determining components of winter oilseed rape compared with winter wheat. Eur. J. Agron..

[9] Morrison MJ, Stewart DW (2002). Heat stress during flowering in summer Brassica. Crop Sci..

[10] Weymann W, Böttcher U, Sieling K, Kage H (2015). Effects of weather conditions during different growth phases on yield formation of winter oilseed rape. Field Crops Res..

[11] Takashima NE, Rondanini D, Puhl LE, Mirallas DJ (2013). Environmental Factors affecting yield variability in spring and winter rapeseed genotypes cultivated in the south eastern Argentine pampas. Eur. J. Agron..

[12] Hoffmann MP, Jacobs A, Whitbread AM (2015). Crop modelling based analysis of site-specific production limitations of winter oilseed rape in northern Germany. Field Crop Res..

[13] Asare E. *The effects of nitrogen and sulphur fertilizers on the growth, yield and seed quality of oilseed rape* (*Brassica napus* L.) PhD thesis (Wye College, University of London, 1991).

[14] Parker DE, Legg TP, Folland CK (1992). A new daily Central England Temperature Series, 1772–1991. Int. J. Clim..

[15] Hurrell JW (1995). Decadal trends in the North Atlantic Oscillation and relationships to regional temperature and precipitation. Science.

[16] UK Meteorological Office 2012 Met Office Integrated Data Archive System (MIDAS) Land and Marine Surface Stations Data (1853-current). NCAS British Atmospheric Data Centre, http://catalogue.ceda.ac.uk/uuid/220a65615218d5c9cc9e4785a3234bd0 (2013).

[17] Lancashire PD (1991). A uniform decimal code for growth stages of crops and weeds. Ann. appl. Biol..

[18] Cohen J (2014). Recent Arctic amplification and extreme mid-latitude weather. Nat. Geosci..

[19] Overland JE (2016). Nonlinear response of mid-latitude weather to the changing Arctic. Nat. Clim. Change.

[20] Habekotté B (1997). A model of the phenological development of winter oilseed rape. (*Brassica napus* L.). Field Crops Res..

[21] Evans JG (2016). Soil water content in southern England derived from a cosmic-ray soil moisture observing system – COSMOS-UK. Hydrol. Process..

[22] Bennett EJ (2017). Development of a Statistical Crop Model to Explain the Relationship between Seed Yield and Phenotypic Diversity within the Brassica napus Genepool. Agronomy.

[23] Kettlewell PS, Sothern RB, Koukkari WL (1999). UK wheat quality and economic value are dependent on the North Atlantic Oscillation. J. Cereal Sci..

[24] Wilby RL, O’Hare G, Barnsley N (1997). The North Atlantic Oscillation and British Isles climate variability. Weather.

[25] Post E, Stenseth NC (1999). Climate variability, plant phenology and northern ungulates. Ecology.

[26] Briers RA, Gee JHR, Geoghegan R (2004). Effects of the North Atlantic Oscillation on growth and phenology of stream insects. Ecography..

[27] Westgarth-Smith AR, Roy DR, Scholze M, Tucker A, Sumpter JP (2012). The role of the North Atlantic Oscillation in controlling U.K. butterfly population size and phenology. Ecol Entomol..

[28] Elliott JM, Hurley MA, Maberly SC (2000). The emergence period of sea trout fry in a Lake District stream correlates with the North Atlantic Oscillation. J. Fish Biol..

[29] Delworth TL (2016). The North Atlantic Oscillation as a driver of rapid climate change in the Northern Hemisphere *Nat*. Geoscience.

[30] Knight, S. *et al*. Desk study to evaluate contributory causes of the current ‘yield plateau’ in wheat and oilseed rape. HGCA project report No. **502** (2012).

[31] Atkinson MD, Kettlewell PS, Poulton PR, Hollins PD (2008). Grain quality in the Broadbalk Wheat Experiment and the winter North Atlantic Oscillation. J. Agric. Sci..

[32] Donatelli M, Srivastava AK, Duveiler G, Niemeyer S, Fumagalli D (2015). Climate change impact and potential adaptation strategies under alternate realizations of climate scenarios for three major crops in Europe. Env. Res. Lett..

[33] He Y, Revell BJ, Leng B, Feng Z (2017). The effects of weather on oilseed rape (OSR) yield in China: future implications of climate change. Sustainability.

