# Supplementary material for: Yield instability of winter oilseed rape modulated by early winter temperature
Source: Sci Rep. 2019 May 6;9:6953. doi: 10.1038/s41598-019-43461-7 (PMC6502865; doi:10.1038/s41598-019-43461-7)
Supplement: Supplementary file 1 — Supplementary Figures and Table [file 41598_2019_43461_MOESM1_ESM.docx]

**Yield instability of winter oilseed rape modulated by early winter temperature**

**James K.M. Brown, Rebecca Beeby and Steven Penfield**

**Supplementary Information**


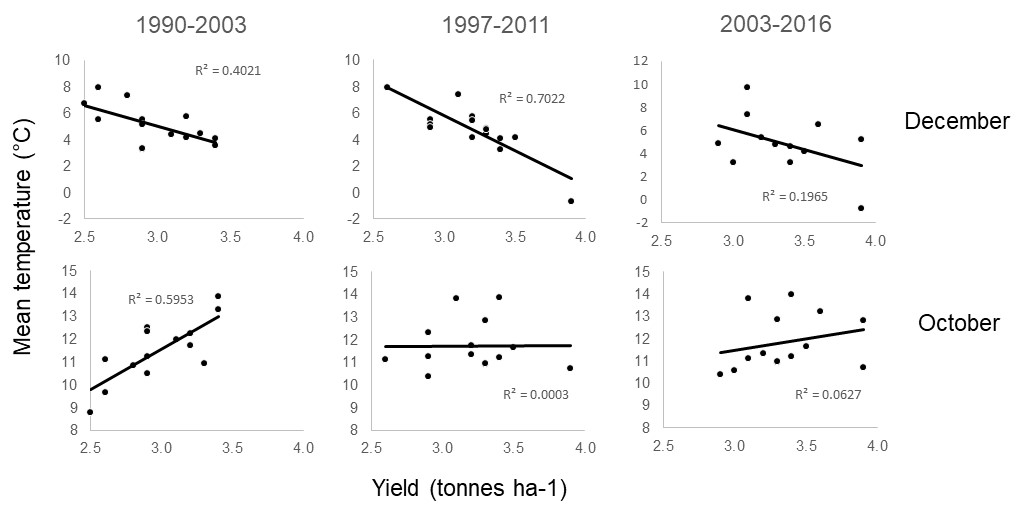


**Figure S1. Changes in the relationship between October and December temperature and UK WOSR yield.** Data shows mean on-farm yields the mean HadCET temperature value for each harvest year for the time ranges indicated.

**
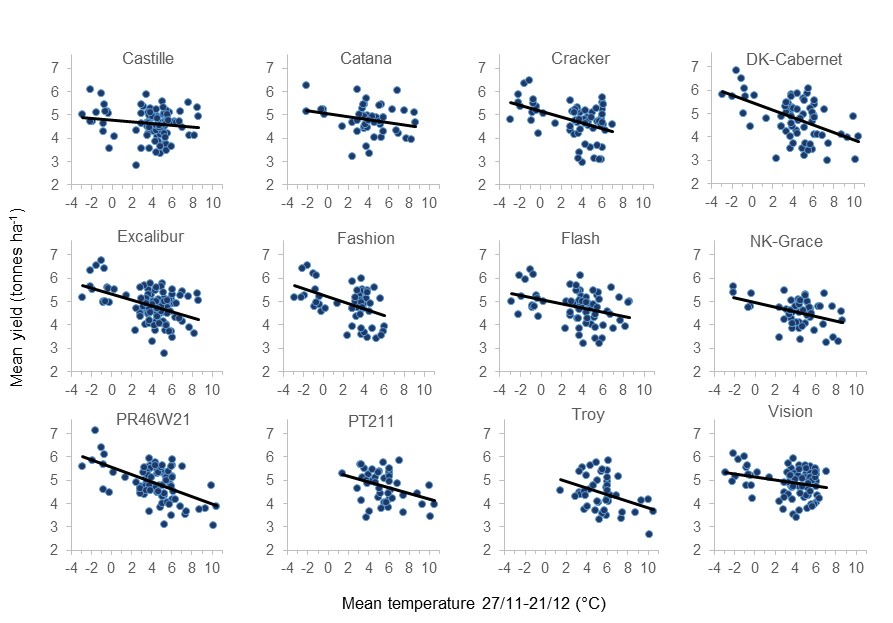
**

**Figure S2. Yield versus December mean trial temperature relationships for selected individual varieties.** Charts the varieties with the highest (DK Cabernet) and lowest (Castille) yield sensitivity. Data points represent trial mean yields for each variety (2 plots per variety per trial).


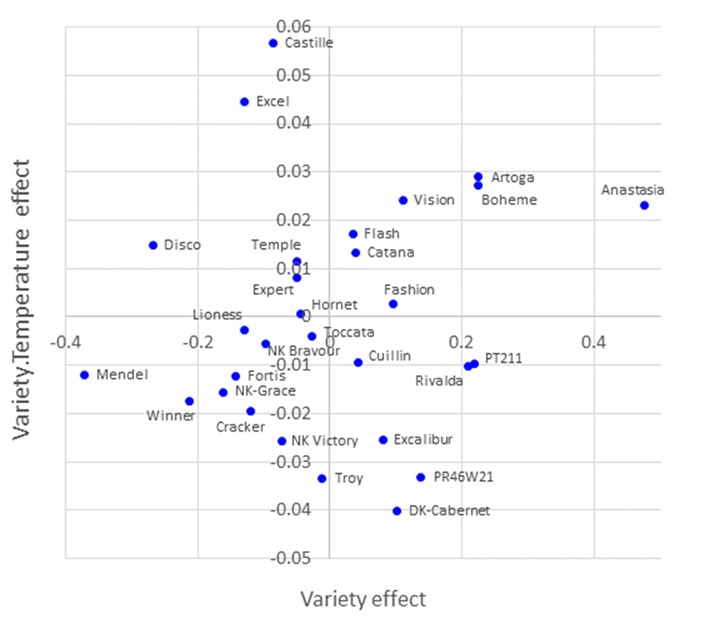


**Figure S3. Relationship between yield and yield stability in UK WOSR.** There is no significant correlation between the yield (t ha^−1^) of each variety (Variety effect) and the yield stability (t ha^−1^ °C^−1^), **i**ndicated by the model-predicted Variety.Temperature effect (R2 = 0.017; P = 0.93). A positive Variety effect indicates that the variety has a higher than average estimated mean yield across the trial series. A positive Variety.Temperature effect indicates that the variety’s yield declines less than the average as December temperature increases.


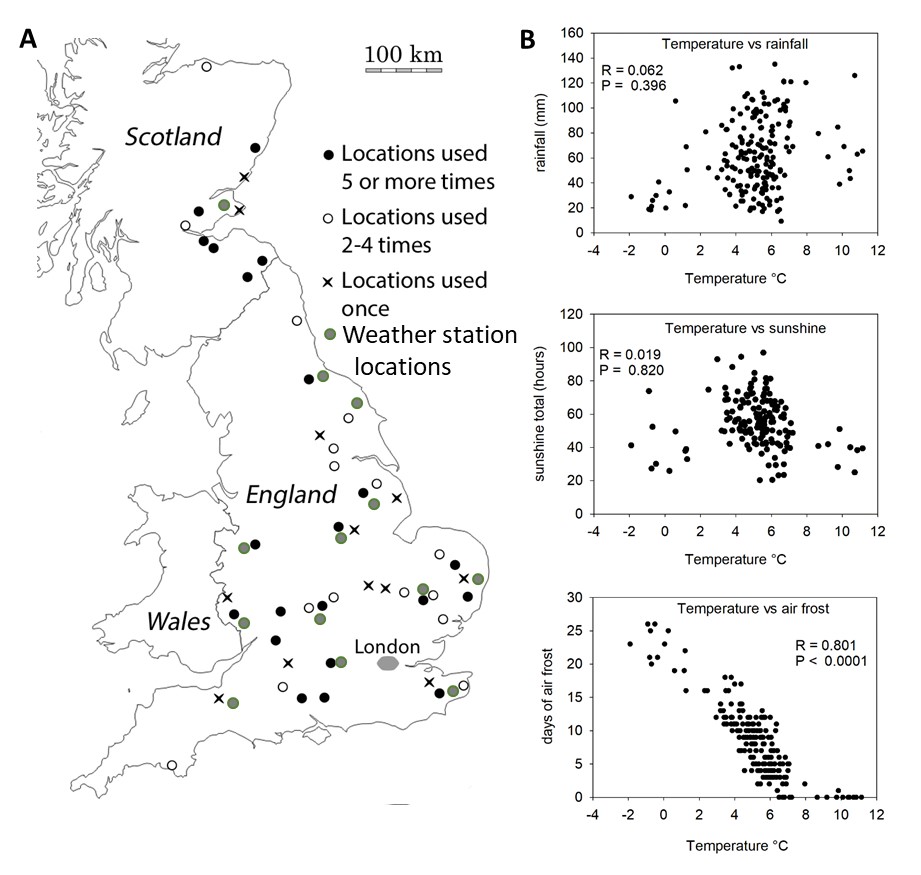


**Figure S4. Testing for aliasing between temperature, rainfall and sunshine in December in the United Kingdom between 2002 and 2016.** Weather data was analysed at 12 sites in major UK winter rapeseed growing areas, including monthly average temperature, total rainfall, sunshine hours and days of air frost (https://www.metoffice.gov.uk/public/weather/climate-historic/#?tab=climateHistoric). No significant relationship was observed between temperature and rainfall across sites (P = 0.4), or between temperature and total sunshine hours (P = 0.82). Mean temperature is strongly correlated with the number of days of air frost (P < 0.0001). We conclude that across sites relationships between mean temperature and yield are not likely due to confounding effects of rainfall or solar radiation. However, we cannot rule out a promotive effect of air frosts on rapeseed yields. Map was created in Microsoft Powerpoint for Office 365 MSO (https://products.office.com/en-gb/powerpoint).

**Supplementary Table 1.** Locations of AHDB Recommended List trials of winter oilseed rape from 2001/02 to 2015/16. Site names are those recorded in the RL trial archives (<https://cereals.ahdb.org.uk/varieties/current-trials-and-harvest-results/archive.aspx>). Trials at nearby sites, on similar land and under the same management were grouped into Locations.

| **Location** | **Site names** | **County/Area** | **Country** | **Harvest years** | **Trials** |
| --- | --- | --- | --- | --- | --- |
| Aby | Aby | Lincolnshire | England | 2012 | 1 |
| Alkerton | Alkerton | Oxfordshire | England | 2012, 2014, 2015 | 3 |
| Anstruther | Anstruther | Fife | Scotland | 2012 | 1 |
| Arbroath | Arbroath | Angus | Scotland | 2009 | 1 |
| Ashford | Ashford, Wye | Kent | England | 2002, 2004, 2005, 2009, 2012 | 5 |
| Berwickshire | Coldstream, Duns, Whitsome | Scottish Borders | Scotland | 2002-2008 | 7 |
| Bingham | Bingham, Cropwell Butler | Nottinghamshire | England | 2002-2014 | 13 |
| Broughton | Broughton | Hampshire | England | 2007, 2009, 2010, 2013, 2015, 2016 | 6 |
| Callow | Callow | Herefordshire | England | 2004-2006, 2008-2016 | 12 |
| Cirencester | Cirencester | Gloucestershire | England | 2002-2006 | 5 |
| Cliffe | Cliffe | North Yorkshire | England | 2010, 2011 | 2 |
| Colchester | Horkesley, Wormingford | Essex | England | 2012, 2014 | 2 |
| Cowlinge | Cowlinge | Suffolk | England | 2008-2011, 2013, 2014 | 6 |
| Croft-on-Tees | Croft-on-Tees | North Yorkshire | England | 2009-2012, 2015, 2016 | 6 |
| Deal | Betteshanger, Tilmanstone | Kent | England | 2014, 2016 | 2 |
| E.Dereham | East Dereham, Swaffham | Norfolk | England | 2002, 2003 | 2 |
| E.Howgate | Easter Howgate | Midlothian | Scotland | 2009-2011, 2012, 2014-2016 | 7 |
| Elgin | Elgin | Moray | Scotland | 2004, 2006 | 2 |
| Framlingham | Framlingham, Tannington, Wilby | Suffolk | England | 2002- 2011, 2013, 2014, 2016 | 13 |
| Goring | Goring, Ipsden, South Stoke | Oxfordshire | England | 2003, 2005-2008, 2010, 2011 | 7 |
| Harlaxton | Harlaxton | Lincolnshire | England | 2015 | 1 |
| King'sPyon | King's Pyon | Herefordshire | England | 2002 | 1 |
| Kingston | Kingston | Devon | England | 2002, 2003, 2005 | 3 |
| Knapwell | Knapwell | Cambridgeshire | England | 2002 | 1 |
| L.Staughton | Little Staughton | Bedfordshire | England | 2003 | 1 |
| Laurencekirk | Fordoun, Laurencekirk, Luthermuir | Aberdeenshire | Scotland | 2003-2006, 2008, 2010, 2011, 2013-2016 | 11 |
| Lenham | Lenham | Kent | England | 2011 | 1 |
| LongMarston | Long Marston | North Yorkshire | England | 2002 | 1 |
| Malton | Duggleby, Malton | North Yorkshire | England | 2003-2005, 2007 | 4 |
| Milnathort | Kinross, Milnathort, Perth | Kinrossshire | Scotland | 2002-2008, 2010, 2011 | 9 |
| Morley | Attleborough, Morley, Wymondham | Norfolk | England | 2005, 2007, 2008, 2011, 2013, 2015, 2016 | 7 |
| Morpeth | Morpeth | Northumberland | England | 2002- 2005 | 4 |
| N.Glos | Blockley, Chipping Campden | Gloucestershire | England | 2008-2011, 2015 | 5 |
| Northants | Daventry, Harlestone, Northampton | Northamptonshire | England | 2002, 2003, 2005, 2007 | 4 |
| Owmby | Owmby-by-Spital | Lincolnshire | England | 2005-2008, 2010-2014 | 9 |
| PulhamMkt | Pulham Market | Norfolk | England | 2004 | 1 |
| Rothwell | Caistor, Market Rasen, Rothwell | Lincolnshire | England | 2002, 2003, 2012, 2016 | 4 |
| Roxburghshire | Kelso, St Boswells | Scottish Borders | Scotland | 2010-2012, 2014-2016 | 6 |
| S.Yorks | Moorends, Sykehouse, Thorne | South Yorkshire | England | 2013, 2015, 2016 | 3 |
| Stonehenge | Shrewton, Wylye | Wiltshire | England | 2004, 2005 | 2 |
| Swindon | Swindon | Wiltshire | England | 2003 | 1 |
| Telford | Edgmond, Harper Adams, Newport, Telford | Shropshire | England | 2002-2004, 2006-2009, 2012, 2013, 2015 | 10 |
| Teversham | Teversham | Cambridgeshire | England | 2005, 2006, 2007 | 3 |
| Thurloxton | Thurloxton | Somerset | England | 2002 | 1 |
| W.Fife | Culross, Kincardine | Fife | Scotland | 2013-2016 | 4 |
| W.Lothian | Edinburgh, Kirkliston, Newbridge, Ratho, Wilkieston | Edinburgh & West Lothian | Scotland | 2002-2008 | 7 |
| Wardington | Wardington | Oxfordshire | England | 2009-2016 | 8 |
| Wickhambrook | Wickhambrook | Suffolk | England | 2013, 2014 | 2 |
| Winchester | Itchen Stoke, Stoke Charity, Winchester | Hampshire | England | 2002-2006 | 5 |
